# Prognosis of patients with traumatic intractable intracranial hypertension based on the time at which craniectomy was performed

**DOI:** 10.4103/0974-2700.70768

**Published:** 2010

**Authors:** Luciano Santana-Cabrera, Alina Uriarte-Rodríguez, Lorea Ugalde-Jáuregui, Rosa Lorenzo-Torrent, Manuel Sánchez-Palacios

**Affiliations:** Intensive Care Unit, Universitary Hospital Insular in Gran Canaria, Las Palmas de Gran Canaria, Spain

Sir,

Intracranial hypertension is the most frequent cause of death and disability, after severe traumatic brain injury (TBI). It is treated with first line therapeutic measures such as normothermia, sedation, moderate hypocapnia, mannitol, etc. When these measures fail to control intracranial hypertension, second line therapies such as barbiturates, hyperventilation, moderate hypothermia or decompressive craniectomy (DC) are used.[1] The beneficial effect and the timing of DC, in the treatment of TBI, remains controversial, although some studies have shown that the DC results in good functional outcome in >50% of patients with severe TBI.[2]

We conducted a retrospective and observational single-center study during a period of four consecutive years, at the Intensive Care Unit (ICU) of the University Hospital Insular in Gran Canaria, Canary Islands, Spain. The aim of our study was to compare the prognosis of patients with post-traumatic intractable intracranial hypertension, based on the time at which craniectomy was performed, that is, during the first 24 hours of admission (primary DC) or more than 24 hours (secondary DC) since the trauma occurred.

Demographic data, mechanism of the trauma, APACHE II, Glasgow Coma Score (GCS) at admission, type of injury (based on the National Traumatic Coma Data Bank CT scan classification), associated injuries, length of stay, ICU mortality and hospital mortality, neurologic state based on GCS at ICU and hospital discharge, and the Glasgow Outcome Score (GOS) at ICU discharge and 6 months later, were analyzed.

One hundred and fifty-six patients were admitted at our ICU with a diagnosis of TBI. DC was performed in 36 patients (23%) with post-traumatic intractable intracranial hypertension. DC was done during the first 24 hours since the trauma occurred in 22 patients (14.1%), and in 14 patients (9%), it was done past the first 24 hours. Both the groups were similar regarding the age, sex, APACHE II, initial GCS and the absence of associated injuries. No significant differences were found between both the groups either in the hospital mortality (*P* = 0.11) or in the percentage of patients who recovered favorably (GOS 4–5 after 6 months from hospital discharge; *P* = 0.67) [Table 1]. Therefore, no tendency toward either increased or decreased incidence in favorable outcome was found relative to the time from admission to DC.

**Table 1:** Differences among patients with posttraumatic intractable intracranial hypertension based on the time at which craniectomy was performed after the trauma

	<24 hours (*n* = 22)	≥24 hours (*n* = 14)	*P* value
Mean age ± SD	39.8±17.1	47.8±17.6	0.18
Male, n (%)	17 (77.3)	12 (85.7)	0.51
APACHE II score ± SD	16.7±6.9	15.1±5.1	0.46
Initial CGS ± SD	6.8±3.9	8.7±4.4	0.18
Absence of associated injuries	13 (59.1)	9 (64.3)	0.75
Hospital mortality	10 (45.5)	3 (21>4)	0.11
GOS 4–5 after 6 months	11 (50)	6 (42.8)	0.67

SD: STANDARD DEVIATION; FIGURES IN PARENTHESIS ARE IN PERCENTAGE

Currently, there are no randomized and controlled clinical trials supporting or rejecting the practice of DC in adults; most published reports provide level II of evidence. However, some of these studies have shown that the outcome is better in patients with DC performed early to prevent intracranial hypertension from occurring more than 12 hours.[3] In other studies, the timing of DC showed no clear trend, for either good neurologic outcome or death,[4] and predictive factors as age, midline shift, and status of the basal cisterns on cranial computed tomography (cCT) were associated with the long-term outcome.[5] We conclude that the timing of DC showed no clear trend, for either good neurologic outcome or death.
